# Evaluation of [^99m^Tc][Tc-HYNIC/EDDA]-Tyr as a target for metabolic tumor imaging in B16F10 melanoma tumor 

**DOI:** 10.22038/AOJNMB.2021.60334.1420

**Published:** 2022

**Authors:** Hemat Yaghoubi Mogadam, Mostafa Erfani, Mohammad Nikpassand, Masoud Mokhtary

**Affiliations:** 1 Department of chemistry, Rasht Branch, Islamic Azad University, Rasht, Iran; 2 Radiation Applications Research School, Nuclear Science and Technology Research Institute, Tehran, Iran

**Keywords:** Protein metabolism, Labeled amino acid, Imaging agent, Melanoma

## Abstract

**Objective(s):**

Clinical interest in metabolic imaging of cancer has been growing in recent years. The increase in protein metabolism of cancer cells is interesting target for metabolic tumor imaging, for which radiolabeled amino acids can be applied. The aim of this study was to evaluate a newly developed radiolabeled amino acid as an imaging protein metabolism in melanoma tumor.

**Methods:**

The radiolabeled tyrosine ([^99m^Tc][Tc-HYNIC/EDDA]-Tyr) was prepared and its biological properties was evaluated in B16F10 melanoma tumor. Moreover organs uptake and tumor accumulation were measured in mouse bearing B16F10 melanoma tumor.

**Results:**

Radiolabeled tyrosine was attached in B16F10 melanoma cells and showed the cell binding capacity of 13.82±0.73%. In animal study, the accumulation of radiolabeled tyrosine was observed in B16F10 melanoma tumor (2.15±0.09 %ID/g) after 30 min post injection, so that the uptake ratio of tumor to muscle was about 5.11. Through scintigraphy process the melanoma tumor clearly visualized in mice at 30 min post injection.

**Conclusion:**

These data suggest that the novel radiotracer ([^99m^Tc][Tc-HYNIC/EDDA]-Tyr) as an protein metabolism imaging agent, is able to transfer into melanoma cells and show great expectation for the clinical application in the imaging of melanoma tumors.

## Introduction

 During past years, the clinical interest in metabolic imaging of cancer has increased. The most obvious example is the increasing use of ^18^F-fluoro-2-deoxy-D-glucose (FDG) in many types of cancer. Growth of anaerobic glycolysis, which is present in nearly all cancer cells, is the goal of FDG uptake (1). Another interesting target for metabolic tumor imaging is the increased protein metabolism in cancer cells. In view of that malignant tissue often depends on increased amino acid uptake, radiolabeled amino acids have become an attractive class of compounds to be used as radiotracer to study cancer biology. Also, these amino acid tracers may help in the regions such as the brain, heart,and inflamed areas which use glucose as nutrient; therefore, these regions are difficult todistinguish from metastasis in [^18^F]-FDG studies.

 The passage of amino acids through the cell membrane is an important factor in protein metabolism to maintain cell integrity and cell cycle progression. A number of amino acid transporter systems have been identified (1). The cells transfer most amino acids through some systems such as a Na-dependent transporter system A and system ASC or an energy-independent L-type amino acid transporter system (2). L-type amino acid transporter 1 (LAT1) is a neutral amino acid transport system and is a major route for the transport of large neutral amino acids, including tyrosine, through the plasma membrane. The use of amino acids for energy, protein synthesis, and cell division increases in malignant transformation. Tumor cells have been found to have overexpressed transporter systems (3, 4). LAT1 is upregulated in various cancers, including melanoma, and contributes to supporting cancer cell proliferation. Therefore, LAT1 is regarded as a promising molecular target of novel imaging agents.

 Most amino acids have been radiolabeled with positron-emitting radioisotopes for positron emission tomography (PET) imaging. These radiolabeled amino acids differ with regards to the ease of synthesis, biodistribution and formation of radiolabeled metabolites in vivo. For these reasons, mainly [^11^C-methyl]-methionine and tyrosine have been studied clinically. More recently, artificial analogues of tyrosine amino acid such as L-3-[^123^I]iodo-alpha-methyl-tyrosine (IMT) or L-3-[^18^F]fluoro-alpha-methyl-tyrosine (FMT) (5-8) and O-2-[^18^F]fluoroethyl-L-tyrosine (FET) (9) have been studied. However it would be a great idea to design and prepare compounds with the same structure and labeled with gamma emitters radioisotopes that can be used in most medicine centers.

 Previously, a new tyrosine amino acid conjugate comprising HYNIC as a chelator residue that has been widely used in labeling the biological compounds was synthesized (10). It was designed by conjugation of the L-tyrosine amino acid with a chelator through the amine site while the hydroxy group present in the amino acid did not involve in the chelating and was free. This amino acid conjugate showed the ability to bind to glioma tumor cells (C6) when labeled with gamma emitter radionuclide [^99m^Tc]Tc. The accumulation of radiotracer in glioma tumor through cell studies and easily tumor localization after injection in rat as the observed results suggested further research on it to be considered as a single photon emission computed tomography (SPECT) imaging agent for other tumors.

 Melanoma, which is the most malignant skin cancer type, has got one of the fastest increasing incidence rates of all cancer types in the world. When be lately diagnosed, melanoma is extremely invasive and metastatic, and accordingly the early detection is necessary. In order to investigate further the applications of this new designed radiotracer and since its possibility to be used as a diagnostic agent in melanoma tumor has not yet been investigated, the present study was designed and conducted. So, ([^99m^Tc][Tc-HYNIC/EDDA]-Tyr) as a radio-tracer was prepared and its properties were evaluated to see whether it could be used as an melanoma imaging agent.

## Methods

 HYNIC-Tyr was prepared through solid phase peptide synthesizing method and according to our previously reported procedure (10). All the chemical agents were purchased from the market sources. Sodium pertechnetate solution ([^99m^Tc]NaTcO_4_) was obtained from a commercial [^99^Mo]Mo-[^99m^Tc]Tc generator (Pars Isotope Co). High performance liquid chromatography evaluation was carried out through a system (Sykam S7131) with a gamma (γ) detector (Raytest-Gabi). Radioactive thin layer chromatography analysis was performed through a scanner (Raytest-GITA, Germany).

***Radiolabeling and quality control***

 Radiolabeling was performed by adding the stock solution (5 μg of HYNIC-Tyr; 10 μl), EDDA (5 mg), and tricine (15 mg) to water (0.5 ml). 40 μl stannous chloride dihydrate in 0.1 M HCl (1 mg/ml) was added to the solution. After that saline solution containing [^99m^Tc]technetium pertechnetate (740 MBq/mL) was added to the final solution. The pH was adjusted to 7 by adding 1 M NaOH. The solution was incubated for 15 min at 95°C. The reaction mixture was evaluated by chromatography.

 In instance thin layer chromatography (ITLC), silica gel strips were used as the fixed phase, water: acetonitrile 1:1 related to hydrolyzed-reduced [^99m^Tc]Tc (Rf~0.0), ([^99m^Tc][Tc-HYNIC /EDDA]-Tyr) and [^99m^Tc]Tc-pertechnetate (Rf~1.0) and ethyl methyl ketone for [^99m^Tc]Tc-pertechnetate (Rf~1.0), ([^99m^Tc] [Tc-HYNIC/ EDDA]-Tyr) and hydrolyzed-reduced [^99m^Tc]Tc (Rf~0.0); were used as the mobile phases. ([^99m^Tc][Tc-HYNIC/EDDA]-Tyr) was characteri-zed by reverse phase HPLC coupled to gamma detector. The radiochemical separation was performed in ODS-5 μm (4.6/250) column followed by the gradient solvent system of acetonitrile/water from ratio of 5%–100% acetonitrile during 25 min (flow rate of 1 mL/min). In vitro stability was evaluated according to above HPLC and RTLC methods using a sample of radiotracer (100 μl) stored in NaCl 0.9% (w/v) (900 μl) and in human serum (900 μl) during 1, 4, 6, and 24 hr after labeling through the previously reported method (11).

***Cell culture and in vitro cell uptake***

 B16F10 murine melanoma cells were obtained from Pasteur Institute of Iran and cultured in RPMI1640 supplemented with 10% fetal bovine serum (FBS) and incubated at 37°C in a humidified atmosphere of 5% CO2. The cells were trypsinized (0.025% trypsin/0.01% EDTA)

and after centrifugation (500 rpm for 3 min) resuspended in PBS. The tubes containing cell suspension (1.5×106 cells/ml) were prepared. After that 100 μl (592 nmol, 370 KBq) of radiotracer was added to the tubes. In a parallel experiment, the blocking test was carried out in presence of excess amino acid tyrosine (500-fold, 296 μmol). The experiment was performed in triplicate for unblock and block cell suspension. The tubes were incubated at 37°C for 1, 2 and 4 hours, and after that time the tubes were centrifuged at 500 g for 3 min and washed with PBS to separate radioactivity associated with the cells from the unbound activity. Afterward, the supernatants of each tube were removed and the cell bound and free radiotracer activities were measured in a γ-counter.

***Biodistribution in melanoma tumor induced mice***

 Animal studies were carried out in accordance with the regulations of our institution and with generally accepted guidelines governing such work. C57 female mice with six weeks old from Pasteur Institute of Iran) were used for biodistribution study. 50 μL of B16F10 cell suspension (1 ×10^7^ cells) prepared as an above mentioned procedure was injected subcutaneously on the left shoulder of each mouse. The tumors were developed after 10 days. 50 µL (3.7 MBq) of radiotracer solution was injected through the tail vein. To define the absorption characteristics of the radiotracer, a group of mice (blocked) received an additional amount (1 µmol) of tyrosine amino acid instantly before injecting the radiotracer. The animals were sacrificed at 30, 1, 2 and 4 hours after injection. Blood and organs such as kidneys, liver, intestine, lungs, tumor, spleen, heart, stomach, thyroid and bone were excised and weighed, and the amount of reactivity with each tissue was counted through a well type gamma counter.

***Imaging***

 In vivo imaging studies were performed for determination of whole body localization of the radiotracer and tumor targeting. For this purpose, radiotracer (3.7 MBq, 50 µL) was injected to B16-F10 tumor-bearing C57 mice and after specified time, mice were anesthetized by ketamine which was injected intraperitoneally. Then whole body images were obtained at 30 min after injection. Imaging was performed using a single head gamma camera (small area mobile, 140 keV, Siemens, Germany) equipped with high sensitivity parallel whole collimator. For image acquisition, a 10% acceptance window around the 140 keV photo peak was used.

## Results

***Radiotracer Preparation and analysis***

**Figure 1:**
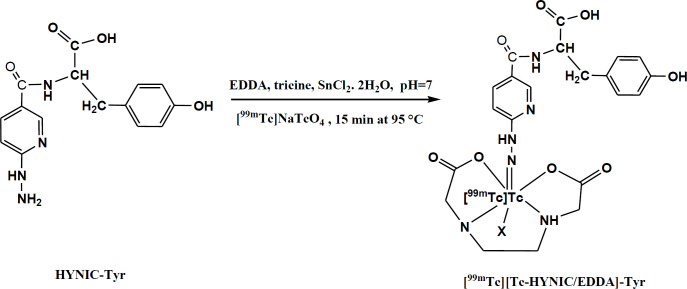
The schema for labeling tyrosine as a target for metabolic tumor imaging and proposed structure for [^99m^Tc][Tc-HYNIC/EDDA]-Tyr as a prepared radiotracer

 Conjugate HYNIC-Tyr was labeled with radionuclide [^99m^Tc]Tc at elevated temperature (95°C, 15 min). Tin(II)chloride was used as the reducing agent and [^99m^Tc]Tc labeling was performed using EDDA as coligand (Figure 1). 

**Figure 2:**
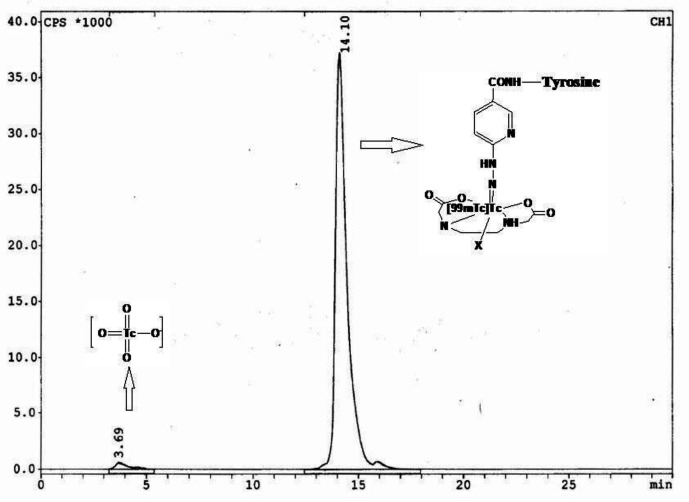
RadioHPLC chromatogram showing the radiochemical purity of the of [^99m^Tc][Tc-HYNIC/EDDA]-Tyr. The values on the x-axis indicate the retention time in minutes. Free pertechnetate eluted earlier than 5 min and radiotracer eluted at 14.10 min

 The radiolabeling yield was >%95 (n=3) while the specific activity was around 123 GBq/μmol. The prepared radiotracer ([^99m^Tc][Tc-HYNIC/ EDDA]-Tyr) was distinguished by HPLC injection. In gamma detector chromatogram, a main peak at retention time of 14.10 minutes followed by a small peak at retention time of 3.69 min were observed which were related to radiotracer and free pertechnetate respectively (Figure 2). 

**Figure 3:**
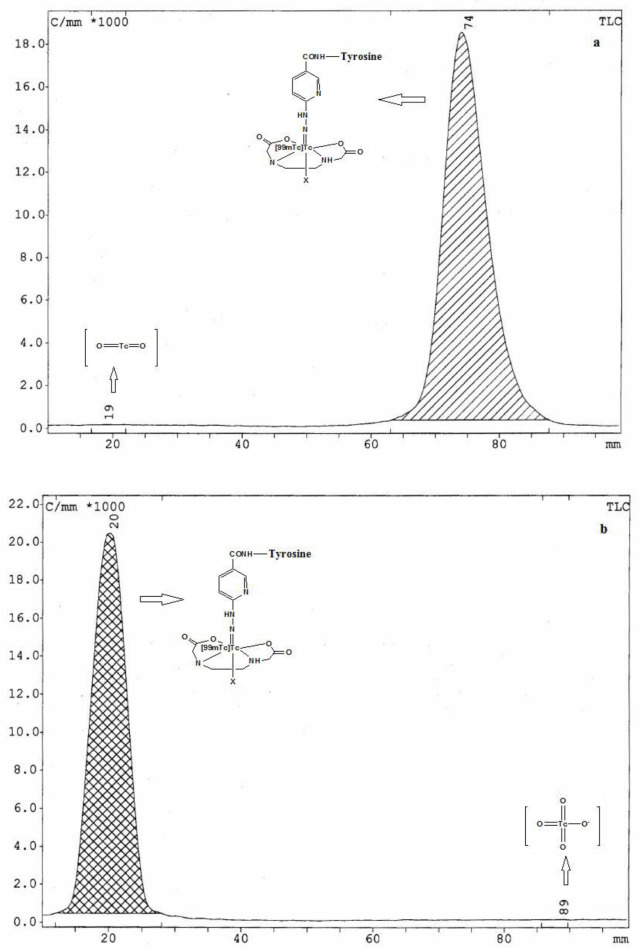
RadioTLC chromatograms of [^99m^Tc][Tc-HYNIC/EDDA]-Tyr in water: acetonitrile 1:1 as a mobile phase radiotracer with Rf~1.0 (**a**) and ethyl methyl ketone as a mobile phase (radiotracer with Rf~ 0.0 (**b**)

 The RTLC results showed that the radiotracer was prepared at a high percentage of radiochemical yield (>%98) and no further treatment was required. Other radiochemical impurity including [^99m^Tc]TcO_4_^-^ (<%1) and reduced hydrolysis of [^99m^Tc]TcO_2_ (<%0.5) 

were negligible (Figure 3). The radiotracer maintained its stability so that after 24 hours in saline and serum media, 97% and 91% of the activity remained with the tracer, respectively (Table 1).

**Table 1:** The stability of a sample of radiotracer in different medium (saline solution and human serum) at different time post labeling

**Time post preparation (h)**	**Stability (%) in saline solution**	**Stability (%) in human serum**
1	98.6±0.3	93.5±0.6
4	98.0±0.2	92.8±1.2
6	97.8±0.4	92.4±1.5
24	97.3±2	91.6±1.1

***Cell uptake study***

**Figure 4:**
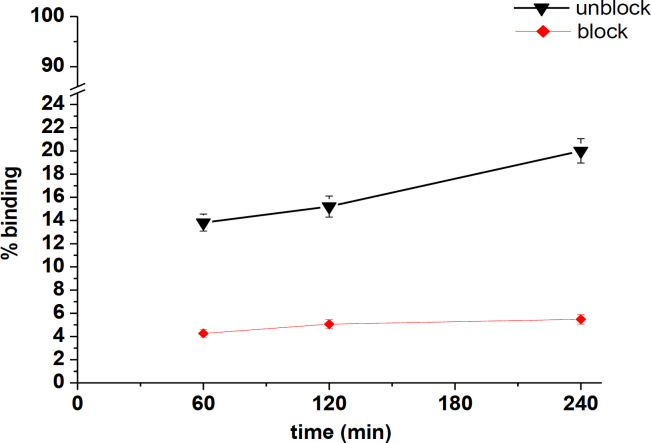
Binding of [^99m^Tc][Tc-HYNIC/EDDA]-Tyr to B16F10 murine melanoma cells. The cells were incubated with [^99m^Tc][Tc-HYNIC/EDDA]-Tyr (100 μl (592 nmol, 370 KBq)) for 1, 2 and 4 hours at 37°C. The cells were blocked in presence of excess tyrosine (500-fold, 296 μmol)

 Binding results of radiotracer specificity to B16F10 murine melanoma cells showed binding capacity of radiotracer (13.82±0.73%, 15.21±0.91% and 20.13±1.05% of the total activity at 1, 2 and 4 hours respectively) (Figure 4). Binding was specific for radiotracer because by adding the excess amount of unlabeled tracer, the binding was reduced to one-third of the original value and was blocked about 3 times. The non-specific binding was 4.27±0.33%, 5.06±0.39% and 5.48±0.41% for 1, 2 and 4 hours.

***Biodistribution***

**Figure 5:**
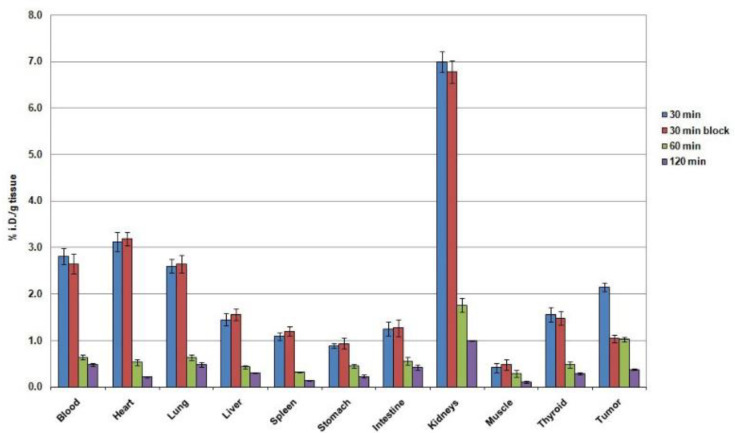
The uptake of radiotracer into tumoral mice at different time point post injection

 Biodistribution information of [^99m^Tc][Tc-HYNIC/EDDA]-Tyr in C57 mice bearing B16-F10 tumor for different organs at various time periods is display in Table 2 and Figure 5. The values are as a percentage of injected doses per gram of the tissue (%ID/g). No side-effects were observed in the mice during the study. The radiotracer was removed from blood circulation with value of 2.81±0.18% of ID/g at 30 min to 0.48±0.04% of ID/g up to 2 hr post injection. Among all the studied organs the amount of radioactivity in muscle was little and insignificant. Kidneys reached to the highest uptake of radioactivity with value of 6.99±0.22% ID/g at 30 min and decreased to 1.06±0.12% of ID/g during 2 hr after injection. The amount of liver uptake was 1.45±0.13% at 30 min which was lower than kidney uptake (6.99±0.22% ID/g) at the same time. These results demonstrate that most of the whole body activity clearance proceeded via the urinary system. The radioactivity uptake in B16F10 tumor was 2.15±0.09% of ID/g at 30 min which decreased to 1.03±0.05% of ID/g after 1 hr after injection. Passing the time this value decreased to 0.38±0.02% of ID/g at 2 hr after injection. The tumor to muscle uptake ratio achieved to 5.11 in 30 min after injection which decreased to 3.45 within 2 hr.

**Table 2:** Biodistribution of [^99m^Tc][Tc-HYNIC/EDDA]-Tyr in mice bearing B16-F10 xenografts tumor (% injected dose/g organ±SD, n=3). For blocking, 1 µmol of tyrosine amino acid was used

**Organ**	**%ID/g**			
	**30 min**	**30 min block**	**60 min**	**120 min**
Blood	2.81±0.18	2.65±0.21	0.64±0.05	0.48±0.04
Heart	3.12±0.20	3.18±0.14	0.53±0.07	0.22±0.02
Lung	2.60±0.15	2.65±0.19	0.64±0.06	0.48±0.05
Liver	1.45±0.13	1.56±0.12	0.43±0.03	0.30±0.01
Spleen	1.09±0.08	1.20±0.10	0.32±0.01	0.14±0.01
Stomach	0.89±0.05	0.94±0.11	0.45±0.04	0.23±0.03
Intestine	1.25±0.15	1.27±0.18	0.56±0.08	0.42±0.06
Kidneys	6.99±0.22	6.78±0.24	1.76±0.15	1.06±0.12
Muscle	0.42±0.10	0.48±0.12	0.29±0.08	0.11±0.03
Thyroid	1.56±0.16	1.48±0.15	0.48±0.06	0.29±0.02
Tumor	2.15±0.09	1.04±0.08	1.03±0.05	0.38±0.02
Tumor/Blood ratio	0.76		1.60	0.79
Tumor/Muscle ratio	5.11		3.55	3.45
Tumor/Liver ratio	1.48		2.39	1.26
Tumor/Kidneys ratio	0.30		0.58	0.35

 For the group of mice receiving high doses of tyrosine, absorption in the tumor region significantly reduced, while this reduction was not found in other tissues. So that, using excess amino acid tyrosine followed by radiotracer injection a 51.7% reduction in tumor uptake was observed.

***Imaging studies***

 An ordinary whole body image of mice with 

**Figure 6:**
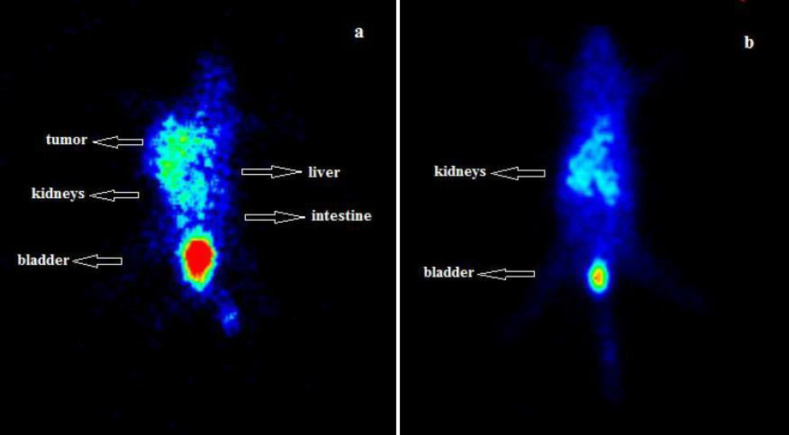
Posterior whole body gamma image of [^99m^Tc][Tc-HYNIC/EDDA]-Tyr distribution in B16-F10 tumor-bearing mice (**a**) and normal mice (**b**). Imaging data is presented at 30 min post-injection of 50 µL (3.7 MBq) radiotracer

B16f10 tumor in shoulder at 30 hr after injection of [^99m^Tc][Tc-HYNIC/EDDA]-Tyr has been shown in Figure 6. The scintigraphy studies indicated that the radiotracer accumulated mainly in kidneys which confirmed its urinary excretion. In early time point, due to rapid renal excretion and low background activity in abdominal regions, the tumor site was clearly recognizable, indicating uptake by melanoma tumor.

## Discussion

 Labeled amino acid tracers can play an important role in tumors imaging of areas where the use of FDG is limited. (12). Formerly, we planned and make by synthesis an amino acid tyrosine analogue conjugated with HYNIC as a bifunctional chelator so that hydroxy group in benzene ring was free and labeled it with technetium-99m (10). The evaluation of its binding to C6 glioma cell revealed specific amino-acid mediated uptake of radiotracer (10). As melanoma often spreads to lymph nodes, lungs, liver and brain, its early detection is important and treatment success is directly related to the size and depth of the cancerous growth. This investigation progressed according to the hypothesis that the radiotracer could be useful in imaging the melanoma tumor where LAT1 that is upregulated in various cancers, including melanoma, has been included.

 Via accompanying ligand, radiotracer ([^99m^Tc][Tc-HYNIC/EDDA]-Tyr) was promptly come into possession. According to chromato-graphic analysis, most of the radioactivity was associated with tracer and the contribution of radioactivity to impurities was negligible, indicating proper formulation and selection of the proper ligand and components for labeling. The right amount of cell binding (13.82±0.73% at 1 h), which is significantly blocked and reduced (about 69%) in the existence of the amino acid tyrosine, indicates the amino acid mediated uptake and the specificity of the radiotracer binding to the melanoma cells. L-type amino acid transporter 1 (LAT1) preferentially transports most of the essential amino acids and is known to be upregulated in a wide spectrum of primary tumors and metastatic lesions from over 20 tissue/organ origins (3). Furthermore, correlations between the LAT1 expressions with poor prognosis have been indicated in various tumors (13-15) and that LAT1 is suitable as a target for radionuclide therapy (16, 17). Considering the above-mentioned facts, it can be argued that amino acid tyrosine based radiotracer structure can play a meaningful role in the observed binding of the compound to the target cell. This binding can provide the necessities for the use of this radiotracer as a diagnostic agent.

 Via this investigation the in vivo biodistribution in C57 mice followed by B16F10 melanoma tumor accumulation have been studied and according to the biodistribution results it can be said that the radiotracer ([^99m^Tc][Tc-HYNIC/EDDA]-Tyr) illustrate extremely favorable biological distribution and with store in the tumor and removal through urinary tracts. In this way, low uptake level for liver showed that the elimination through bile was a minor route. High absorption ratio for tumor/muscle in primary times (5.11 and 3.55 at 30 min and 1 hr after injection, respectively) shows the absorption properties for the tumor area. As well as, the durability of this ratio over time passing (3.45 at 2 hr after injection) can be relevance to the entry of radiotracer within the tumor cells.

 The significant reduction in tumor uptake for blocked comparison with nonblocked experiment (1.04±0.08% of ID/g versus 2.15±0.09% of ID/g, respectively) elucidated the absence of radiotracer accumulation in the tumor unrelated to receptor binding and confirmed the specificity of the radiotracer accumulation in the tumor. These findings can verify that radiotracer have ability to use for imaging of melanoma tumor especially in early stage and after labeling with promising radionuclides could be used as a therapeutic agent.

 For this radiotracer , in rat induced C6 glioma tumor, the tumor/muscle ratios of 5.43, 5.58 and 8.90 at 30 min, 1 hr and 2 hr after injection have been reported respectively (10). Also, the previous study proved the advantage of this radiotracer for its good sensitivity in detection of glioma tumor (10). In comparison, in mice with B16F10 melanoma tumor this radiotracer showed lower tumor/muscle ratios (5.11 versus 5.43, 3.55 versus 5.58 and 3.45 versus 8.9 at 30 min, 1 hr and 2 hr after injection, respectively). On the other hand, this lower ratio can be mediated through the use of melanoma cell lines, which can represent lower values of LAT1 compare to C6 glioma cells. 

 It should be noted that in order to see the tumor area, the sufficient number of molecules of the radiotracer are attached to tumor cells is needed. Therefore, despite the lower tumor uptake in B16F10 melanoma tumor cells compare to rat C6 glioma tumor (2.15±0.09% of ID/g versus 2.61±0.09% of ID/g (10)) there were enough receptors for radiotracer accumulation and to see the tumor clearly in the image. Accordingly, it can be expected that this radiotracer can be used in the detection and diagnosis of melanoma tumors or after conjugation with therapeutic radionuclides, these tumors can be destroyed and treated.

## Conclusion

 A protein metabolism imaging agent was prepared easily with high yield. B16F10 melanoma cell evaluation resulted approve of radiotracer binding. The radiotracer showed effective tumor uptake and high tumor to normal organ ratios. As a consequence, [^99m^Tc][Tc-HYNIC/EDDA]-Tyr seems to be a great nominate for SPECT imaging of tumors and is a favorable choice for targeted radionuclide therapy through further modification and labeling with thera-peutic radionuclides.
